# Defined conditions for propagation and manipulation of mouse embryonic stem cells

**DOI:** 10.1242/dev.173146

**Published:** 2019-03-26

**Authors:** Carla Mulas, Tüzer Kalkan, Ferdinand von Meyenn, Harry G. Leitch, Jennifer Nichols, Austin Smith

**Affiliations:** 1 Wellcome-MRC Cambridge Stem Cell Institute, University of Cambridge, Gleeson Building, Tennis Court Road, Cambridge CB2 1QR, UK; 2 Department of Medical and Molecular Genetics, King's College London, London SE1 9RT, UK; 3 MRC London Institute of Medical Sciences (LMS), Du Cane Road, London W12 0NN, UK; 4 Institute of Clinical Sciences (ICS), Faculty of Medicine, Imperial College London, Du Cane Road, London W12 0NN, UK; 5 Department of Physiology, Development and Neuroscience, University of Cambridge, Downing Street, Cambridge CB2 3DY, UK; 6 Department of Biochemistry, University of Cambridge, Hopkins Building, Tennis Court Road, Cambridge CB2 1QW, UK

**Keywords:** Differentiation, Embryonic stem cells, Pluripotency, Self-renewal

## Abstract

The power of mouse embryonic stem (ES) cells to colonise the developing embryo has revolutionised mammalian developmental genetics and stem cell research. This power is vulnerable, however, to the cell culture environment, deficiencies in which can lead to cellular heterogeneity, adaptive phenotypes, epigenetic aberrations and genetic abnormalities. Here, we provide detailed methodologies for derivation, propagation, genetic modification and primary differentiation of ES cells in 2i or 2i+LIF media without serum or undefined serum substitutes. Implemented diligently, these procedures minimise variability and deviation, thereby improving the efficiency, reproducibility and biological validity of ES cell experimentation.

## Abstract (precis)

**Summary:** Detailed protocols for the propagation, manipulation and differentiation of mouse embryonic stem cells, with tips for minimising variability and improving the efficiency, reproducibility and reliability of embryonic stem cell culture.

## INTRODUCTION

Mouse embryonic stem (ES) cells are cell lines derived from the pre-implantation epiblast of mouse embryos (Boroviak et al., 2014; Brook and Gardner, 1997; Evans and Kaufman, 1981; Martin, 1981). Under appropriate culture conditions they retain the properties of their tissue of origin and can re-enter normal development when introduced into morula- or blastocyst-stage embryos (Bradley et al., 1984). Moreover, ES cells can readily be genetically modified and clonally expanded. Advances such as genome editing using CRISPR/Cas9 have expanded the opportunities for multiplex and/or complex genome engineering in ES cells (Andersson-Rolf et al., 2017; Yang et al., 2013). Their properties make ES cells uniquely powerful tools, both for generating genetically modified mice, and for experimental dissection of fate choice in pluripotent cells. These attributes depend entirely, however, upon the genetic and phenotypic fidelity of ES cells during propagation.

ES cells were first derived in 1981 by culturing early mouse embryos in conditions optimised for teratocarcinoma stem cells (Evans and Kaufman, 1981; Martin, 1981). The capacity of ES cells to contribute to chimaeras, colonise the germline and engender healthy offspring was demonstrated in 1984 (Bradley et al., 1984), establishing that they are non-transformed and, in all essential aspects, genetically normal. This finding provoked a major effort to introduce targeted genetic modifications into mice by implementing homologous recombination in ES cells. It was not until the 1990s, however, that the technology became relatively routine (for a review, see Capecchi, 2005). A major reason for the time gap is that ES cell cultures were frequently found to be aneuploid, particularly after clonal selection. This problem gradually diminished as appreciation spread of the relatively fastidious demands of ES cell culture compared with other cell types (Robertson, 1987). In particular, ES cells must be passaged frequently to avoid any overgrowth, which confers advantage to genetically abnormal cells.

The original culture conditions for ES cells comprised co-culture with a feeder layer of mitotically arrested embryonic fibroblasts and medium containing carefully screened foetal calf serum (FCS) (Robertson, 1987). This effective but complex system was simplified with the discovery that a major contribution of feeders is to provide the cytokine leukaemia inhibitory factor (LIF) (Smith et al., 1988; Williams et al., 1988). Addition of LIF increases the robustness of ES cell cultures on feeders and this remains a widely used system. LIF can also support ES cell derivation and propagation without feeders in either serum or bone morphogenetic protein (BMP) (Nichols et al., 1990; Ying et al., 2003a). In these conditions, however, the cultures are morphologically heterogeneous. Moreover, the cells show fluctuating expression of several transcription factors known to be expressed in the pre-implantation epiblast and downregulated during peri-implantation development (Chambers et al., 2007; Hayashi et al., 2008; Toyooka et al., 2008). Thus, ES cells in these culture conditions do not correspond to a discrete stage of embryonic pluripotency and the developmental relevance of metastable gene expression remains unclear (Filipczyk et al., 2015; Nichols and Smith, 2012; Smith, 2017). In contrast, ES cells appear morphologically and molecularly relatively homogeneous when maintained in defined medium in which the Erk1/2 signalling pathway is blocked and glycogen synthase kinase 3 is partially inhibited (Wray et al., 2010; Ying et al., 2008). Under this dual inhibition, known as 2i, ES cells exhibit transcriptome similarity to pre-implantation epiblast (Boroviak et al., 2014). Importantly, unlike other conditions, use of 2i (or its predecessor 3i) enables reliable derivation of authentic ES cells from different strains of mice and also from another species, the rat (Buehr et al., 2008; Kiyonari et al., 2010; Li et al., 2008; Nichols et al., 2009a).

**Fig. 1.**
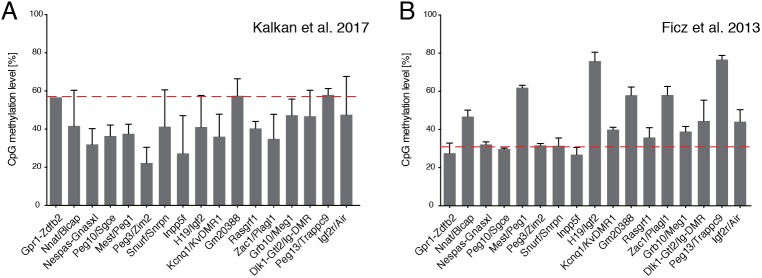
**Imprinted control region (ICR) methylation levels in mouse ESCs.** (A,B) Average CpG methylation levels at known ICRs were quantified in whole genome bisulphite sequencing (WGBS) data sets from an ES cell line maintained in 2i (no LIF) (Kalkan et al., 2017; GEO accession number GSE92273) (A) and from an ES cell line maintained in 2i/LIF (Ficz et al., 2013; GEO accession number GSE42923) (B). Both datasets are derived from inbred 129 strain lines. The WGBS datasets were processed as described previously (von Meyenn et al., 2016) and the mean±s.d. of three experiments are shown. The mean global CpG methylation levels in each condition are shown (red dashed line). These observations are in agreement with previous reports that methylation at three DMRs is maintained in an ES cell line derived and maintained in 2i/LIF and an allele-specific assay confirmation of normal methylation pattern at the same regions in 2i/LIF-derived embryonic germ cells (Leitch et al., 2013a).

We have proposed that the defined culture system using the highly specific 2i inhibitors corrals ES cells in a stable ‘ground state’ (Martello and Smith, 2014). The 2i platform has been widely exploited to study signalling, gene regulation and network control of naive pluripotency, and the transition path from pluripotency to lineage commitment (Blaschke et al., 2013; Carey et al., 2014; Dunn et al., 2014; Ficz et al., 2013; Habibi et al., 2013; Hackett et al., 2017; Hayashi et al., 2012, 2011; Kalkan et al., 2017; Kumar et al., 2014; Leitch et al., 2013a; Marks et al., 2012; McEwen et al., 2018 preprint; Mulas et al., 2017; Murakami et al., 2016; Semrau et al., 2017). Importantly, male ES cells maintained in 2i can retain a euploid karyotype and germline chimaera competency over multiple passages with similar efficiency to cells cultured in serum (Ying et al., 2008; Nichols et al., 2009a; Kiyonari et al., 2010; Leitch et al., 2010, 2013b,a; Leeb et al., 2012; Morgan et al., 2013; Jakubczik et al., 2016; Kalkan et al., 2017; Zhang et al., 2018). Interestingly, ES cells show lower global DNA methylation in 2i than in serum (Ficz et al., 2013; Habibi et al., 2013; Leitch et al., 2013a). Female ES cells are vulnerable to severe hypomethylation (Choi et al., 2017a) and loss of methylation imprints in either 2i or serum, which can reduce their chimaera contribution (Yagi et al., 2017). Male ES cells on the other hand can retain relatively normal methylation imprints (Fig. 1), although loss of specific differentially methylated regions (DMRs) has also been reported in both serum and 2i cultures (Choi et al., 2017b; Dean et al., 1998). Genetic background may be one component influencing loss of imprinted DMRs. Another important factor may be levels of culture stress or stimulation due to differences in media composition and cell handling between laboratories, even when using apparently similar conditions. As with any cells *in vitro*, ES cells are inherently liable to acquire genetic and epigenetic adaptations or abnormalities if subjected to untoward selective pressures. Vulnerability to environmental stress may be enhanced in serum-free culture. Consequently, apparently minor variations in media formulation or culture procedures may give rise to discrepant findings. Reports of subpopulations and cell cycle heterogeneity in 2i culture (Kolodziejczyk et al., 2015) may likewise be influenced by particular culture practices.

The field could benefit, therefore, from a stringent methodology for ES cell culture. To that end, we detail here standardised media composition and cell handling procedures for robust propagation and genetic manipulation of mouse ES cells using 2i in defined medium.

## RESULTS AND DISCUSSION

### Propagation of ES cells without serum factors or feeders

#### Aim

The aim of this procedure is to expand undifferentiated cells in 2i or 2i/LIF (Ying et al., 2008). The anticipated outcome is actively growing colonies of uniform size that are evenly distributed across the dish, with no signs of differentiation, and that can be passaged by enzymatic dissociation and expanded continuously. Note that in defined medium cells are generally less tolerant to suboptimal media conditions or environmental perturbations than in the presence of serum, serum substitutes such as KnockOut Serum Replacement (KSR), or feeders. This susceptibility may manifest as spontaneous differentiation and/or cell death. Cells should not be kept out of the incubator any longer than necessary or exposed to varying incubator environments.

#### Materials

Accutase
0.1-0.2% gelatine in PBS or 10 µg/ml laminin in PBS
Wash medium (DMEM/F12 + BSA; see Table S1)
2i or 2i/LIF [hereafter referred to as 2i (±LIF)] in N2B27 (see Table S1 for media formulation and suggested suppliers)
Haemocytometer
Tissue culture treated plates
Falcon tubes
Bench centrifuge
Humidified incubator at 7% CO_2_ and 37°C

#### Protocol: routine passage of ES cells from a 6-well plate

1. Coat plates or wells with 0.1-0.2% gelatine in the incubator for a minimum of 15 min.
2. Pre-warm necessary volume of 2i (±LIF) or other culture medium, wash medium and Accutase to 37°C. Note: avoid leaving 2i medium or Accutase at 37°C for too long.
3. Remove all gelatine from plates or wells and replace with warm medium. Return to the incubator to pre-equilibrate (not necessary but might help survival, especially for ES cells of non-permissive strains, compromised mutants or when plating at low density). Drying the plates is not necessary.
4. Aspirate medium gently but completely from cells and add 0.5 ml of Accutase per well. Avoid drying the cells.
5. Incubate with Accutase (see Table 1) at room temperature for 4-6 min until colonies decompact and detach. Tap plate to ensure detachment and initial dissociation.

**Table 1.**
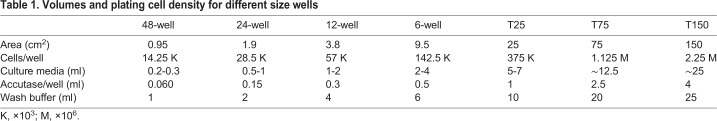
**Volumes and plating cell density for different size wells**
6. Add 1 ml of wash buffer and pipette up and down (without touching the bottom of the dish) 10-20 times in order to obtain a single cell suspension. Try to minimise the formation of bubbles. Tip: check under the microscope to ensure single cell suspension.
7. Transfer cell/Accutase/wash suspension to a Falcon tube containing 6 ml of wash medium (see Table 1).
8. Centrifuge cells at 300 ***g*** for 3.5 min.
9. Aspirate supernatant with care, removing as much liquid as possible without disturbing the pellet.
10. Re-suspend cell pellet by pipetting up and down 10-15 times in 0.5-2 ml of 2i (±LIF) medium, ensuring a single cell suspension is obtained.
11. Count number of cells/ml and plate appropriate number of cells (see Table 1). Slide plate back and forth across a flat surface to distribute cells evenly, then place carefully in incubator. Do not disturb for several hours.

#### Notes

**Fig. 2.**
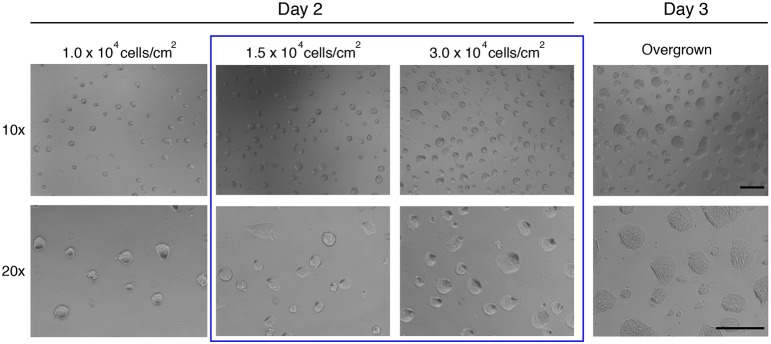
**Representative images of** **ES cells** **in 2i at different densities on day 2 and day 3.** Blue box highlights the range of cell densities ideal for splitting. Note refractile colony edges on day 2, which are lost in overgrown colonies at day 3. Scale bars: 0.5 mm.

Cell density will have a significant effect on metabolism, cell cycle and differentiation kinetics, amongst other factors. This is, therefore, a key parameter to monitor. We have observed that culture at high density or colony overgrowth can compromise ability to differentiate and that this effect may become irreversible. Overgrowth of cells in FCS/LIF conditions is also associated with impaired capacity of differentiation and karyotypic instability, but in defined conditions ES cells must be passaged at smaller colony sizes than in FCS- or KSR-containing medium. A single period of overgrowth may induce a permanent change, even if undifferentiated morphology is retained. Specifically, even if overgrown cells regain refractile domed morphology after passaging, they may be compromised. Two key parameters should be kept relatively constant: (1) the density at which cells are plated after each split; (2) the frequency with which cultures are split. Representative images of cultures ready to be passaged are shown in Fig. 2.

The following cell concentrations are routinely used: for maintenance of cells, 1.5-3.0×10^4^ cells/cm^2^; for most experiments: 1.5×10^4^ cells/cm^2^ (see below for differentiation). See also Table 1. It is highly recommended to count cell numbers at every split to avoid overgrowing cells and to monitor growth rate (see Fig. 2 for representative images).

**Fig. 3.**
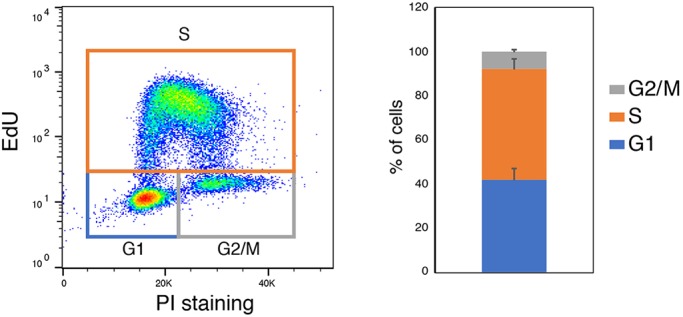
**Typical cell cycle profile of day 2 ES cells plated at 1.5×10^4^ cell/cm^2^.** Cells were stained with propidium iodide (PI) and the Click-iT EdU kit according to manufacturer's instructions. Graph shows quantification over two independent experiments, with two separate lines in each.

In routine culture, cells should be split every 2-3 days (Fig. 2). Healthy cultures double every 12-14 h (Carey et al., 2014). ES cells in 2i have a substantial proportion of cells in G1 phase (Fig. 3) (Huurne et al., 2017). This is in contrast to FCS cultures, in which ∼70% of cells are in S phase (Huurne et al., 2017). Even if plated at lower/clonal density, colonies should not be allowed to grow for more than 4-5 days before passaging.

If passaging is delayed beyond 2 days, medium should be renewed on day 3, and any day thereafter.

The quality of N2B27 must be monitored. Signs of suboptimal N2B27 include flattening of colonies, cells detaching, reduced proliferation rate, or increased cell death.

ES cells from certain genetic backgrounds (e.g.C57BL/6) require 2i/LIF in order to be stably propagated long term, whereas others, such as 129 strains, can be propagated in 2i alone or in single inhibitor with LIF. LIF invariably increases colony formation after low-density plating. The addition of LIF alters the kinetics of differentiation, however, delaying the process by ∼12 h (Nett et al., 2018). Certain mutant cell lines (e.g. those carrying *Etv4/5* gene deletions), are sensitive to MEK inhibition and show more robust proliferation in CH/LIF (LIF containing CHIR99021) (Kalkan et al., 2019). To convert cells between 2i, CH/LIF and PD/LIF (LIF containing PD0325901), two passages over 4-6 days are sufficient. PD/LIF cells typically show a more flattened morphology than those grown in conditions containing CH. Because culture in 2i/LIF is most robust, cells can be kept in 2i/LIF for routine passaging, and transferred to other conditions (e.g. 2i or PD/LIF) for one or two passages before experimental analyses. The cell densities reported above work well for all medium conditions.

Cell lines can either be derived in defined conditions (see below) or adapted from conventional FCS/LIF (with or without feeders) conditions. When converting cells from FCS/LIF, we recommend plating cells first in FCS/LIF and changing media to 2i or 2i/LIF after 24 h. Cell death, mainly associated with elimination of differentiating cells, is often observed for the first few days, but stable homogeneous cultures can be established within one or two passages.

Cells should be routinely maintained without any antibiotics, in keeping with good tissue culture practice (Freshney, 1994). Antibiotics can mask low level microbial infections and may have unknown effects on cell metabolism and gene expression.

Mycoplasma testing should be carried out frequently and on all newly generated or obtained lines using PCR assays and commercial kits. If mycoplasma is detected, the cultures should be discarded (Markoullis et al., 2009).

It is important to maintain two types of cell line stock. Primary stocks are the lowest passage cells available [passage (p) 4-8 for embryo-derived cell lines], that have been genotyped, mycoplasma screened, have at least ∼80% diploid cells (40XY or 40XX) and show uniform morphology. These are kept in liquid nitrogen for long-term storage. Secondary stocks are more numerous at slightly higher passage number (p10-15). These stocks can be kept short term at −80°C and are used for routine experiments. Cells should not normally be passaged beyond 30. However, we have generated high contribution chimeras that give germline transmission after multiple passages of mouse ES cells (p30+), embryonic germ cells (p15+), and haploid ES cells (p20+) in 2i/LIF.

Accutase is routinely used because it is gentler than trypsin, results in a single cell suspension and does not require serum inactivation. TrypLE reagents may also be used, although these occasionally result in incomplete dissociation. Trypsin can be used, but we recommend adding ∼5% FCS to the wash medium (see Table S1) to ensure complete inactivation, or doubling the volume of wash medium, or increasing the BSA concentration.

Attachment of poorly adherent cells, a feature of some mutants and non-129 strains (e.g. CBA, NOD, DBA, etc.), can be improved by plating onto laminin-coated plates. For this, coat plates with laminin solution (∼10 µg/ml in PBS) for a minimum of 2 h at 37°C, before aspirating the solution and adding the culture medium.

DMEM/F12 and Neurobasal buffer can be used at 5-10% CO_2_. We use 7% CO_2_ for compatibility with FCS cultures maintained in GMEM. In the presence of B27 we have not observed any advantage of low O_2_ compared with ambient atmosphere.

### Colony-formation assay

#### Aim

The colony-formation assay is a key functional test in ES cell biology. Cultures are dissociated to a single cell suspension and plated at very low (clonal) density, and the number of colonies that form is determined after several days. This assay can be used as a diagnostic tool for media quality control, and experimentally for determining the proportion of undifferentiated cells in a differentiation or reprogramming study or following a genetic/chemical perturbation, or in a modified culture environment. See Fig. 3 for an example.

#### Materials

Accutase
10 µg/ml laminin in PBS
Wash medium
2i/LIF (see Table S1 for media formulation and suggested suppliers)
FCS+LIF
Haemocytometer
Tissue culture treated plates
Falcon tubes
Centrifuge
Humidified incubator at 7% CO_2_ and 37°C
Alkaline phosphatase staining kit

#### Protocol

1. Coat 12-well plates (provide three wells per sample) with laminin (∼10 µg/ml in PBS) for a minimum of 2 h at 37°C. At clonal density, laminin helps colonies remain attached for the duration of the experiment.
2. Pre-warm Accutase, wash medium, and culture medium (e.g. 2i/LIF).
3. Before starting, aspirate laminin and add 1 ml of 2i/LIF per well. Return plate to the incubator in the meantime to equilibrate medium.
4. Aspirate medium from cells and quickly add appropriate volume of Accutase.
5. Incubate with 1 volume of Accutase at room temperature (RT) for 4-6 min (1 min after the colonies have detached).
6. Add 1 ml of wash medium and pipette up and down 10-20 times in order to obtain a single cell suspension. Tip: check under the microscope to ensure single cell suspension.
7. Transfer cell/Accutase/wash suspension to a Falcon tube containing 5 volumes of wash medium.
8. Centrifuge cells at 300 ***g*** for 3.5 min.
9. Aspirate supernatant with care, removing as much liquid as possible without disturbing the pellet.
10. Re-suspend cell pellet in 0.5-2 ml of culture medium (e.g. 2i/LIF).
11. Count number of cells/ml.
12. Plate 400 cells/well in prepared medium. If necessary, carry out two sequential 1:10 dilutions of the cell to improve accuracy, and aim to pipette ∼50 µl of cell suspension per well.
13. Slide plate back and forth across a flat surface to distribute cells evenly, then place carefully in incubator.
14. After 4-5 days, perform alkaline phosphatase staining according to manufacturer's instructions, let dry, image plate and count the number of colonies.

#### Notes

The use of 12-well or 6-well plates is advisable. Plate 400 cells in 12-well plate, and 800 in 6-well plates. It is recommended to perform at least three technical replicates per condition as high variability can occur when plating small numbers of cells. Fluorescence-activated cell sorting of cells directly onto plates can also be used to reduce technical variability.

Colonies can detach easily; therefore, add fixative gently. Leaving cells for >5 days will increase the likelihood of colony detachment.

**Fig. 4.**
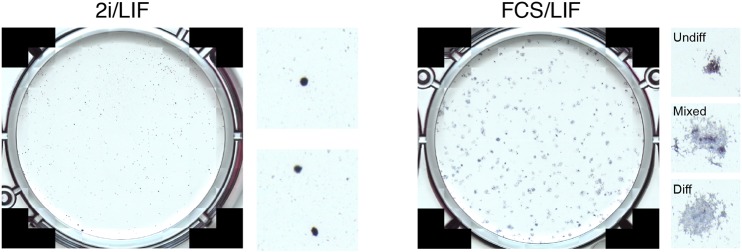
**Representative images of clonal assays in 2i/LIF and FCS/LIF conditions.** Insets to the right show magnified views of individual colonies and suggested classification in the case of FCS/LIF.

We also recommend performing colony-formation assays in parallel FCS/LIF medium and analysing plates by alkaline phosphatase staining on day 3/4. Monitor cultures to make sure colonies do not merge. In FCS/LIF conditions, differentiating cells, which would not survive in 2i/LIF, will persist and contribute to mixed or wholly alkaline phosphatase-negative colonies. This experiment can serve as a control for plating efficiency and differentiation potential. Fig. 4 shows representative images of colony assays in 2i/LIF and FCS/LIF.

Typically, we expect wild-type cells maintained in 2i or 2i/LIF to have a clonogenic capacity of ∼70-80% when plated in 2i/LIF in optimal media (all alkaline phosphatase positive) with minimal differentiation.

### Exit from naïve pluripotency

#### Aim

Examining the kinetics of exit from naïve pluripotency can inform whether a particular perturbation (genetic, chemical or mechanical) accelerates, delays or blocks transition to differentiation (for examples, see Kalkan et al., 2019; Li et al., 2017; Martello et al., 2013; Miller et al., 2016; Nett et al., 2018; Niwa et al., 2009; Wray et al., 2011). This system can also be used for genetic screens to identify regulators of pluripotency progression (Betschinger et al., 2013; Leeb et al., 2014; Li et al., 2018; Villegas et al., 2018; Yang et al., 2012).

#### Materials

Accutase
0.1-0.2% gelatine in PBS or 10 µg/ml laminin in PBS (for timecourse studies longer than 48 h, laminin coating is recommended for better attachment)
Wash medium
2i (±LIF) in N2B27
N2B27 (see Table S1)
PBS
Haemocytometer
Tissue culture treated plates
Falcon tubes
Bench centrifuge
Humidified incubator at 7% CO_2_ and 37°C

#### Protocol: timecourse study over ∼48 h

1. Coat 24-well plates or wells with 0.1-0.2% gelatine in the incubator for a minimum of 15 min. Allow for two or three wells per condition (technical repeats). Use a separate plate for each time point.
2. Pre-warm necessary volume of 2i (±LIF) or other culture medium, wash medium and Accutase to 37°C. Note: avoid leaving 2i medium or Accutase at 37°C for too long.
3. Remove gelatine from plates or wells and replace with warm medium. Return to the incubator to pre-equilibrate (not necessary but might help survival, especially for ES cells of non-permissive strains, compromised mutants or when plating at low density).
4. Split cells as indicated previously, re-suspend cell pellet in 0.5-2 ml of 2i (±LIF) and count number of cells/ml.
5. Plate 30,000 cells per well of a 24-well plate. Slide plate back and forth across flat surface to distribute cells evenly, then place carefully in incubator. Do not disturb for several hours.
6. After 12-24 h, aspirate medium from cells and add 0.5-1 ml of sterile PBS. Aspirate PBS and replace with pre-warmed N2B27. Perform media replacement steps gently to avoid detaching cells.
7. Analyse cells at the appropriate time points.

#### Notes

The exact time at which 2i(±LIF) is replaced by N2B27 to initiate exit from naïve pluripotency is flexible. However, it should be kept constant across experiments. Higher density cultures transition more slowly.

**Fig. 5.**
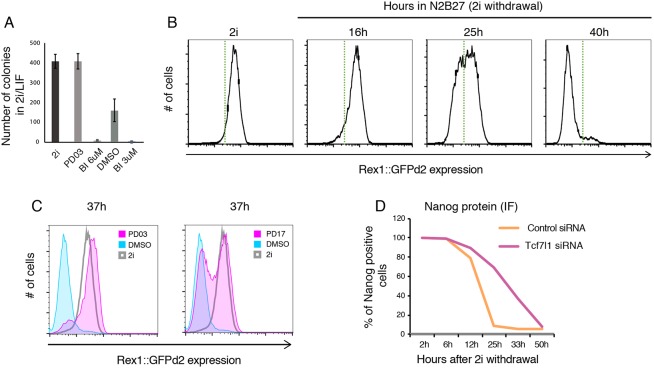
**Example data of exit from pluripotency experiment.** (A) Colony-formation assay. Cells were differentiated for 36 h in four different conditions; PD03, MEK1/2 inhibitor PD0325901 (1 µM); BI, RSK inhibitor BI-D1870 (3 or 6 µM); DMSO, carrier control. (B) Representative downregulation kinetics of Rex1::GFPd2 cells plated in N2B27, starting from 2i (no LIF) conditions. Green dotted line indicates arbitrary threshold. (C) Use of the Rex1::GFPd2 reporter system to determine the effect of two different inhibitors (PD03, MEK1/2 inhibitor PD0325901 at 1 µM; PD17, FGF receptor inhibitor PD173074 at 100 nM) on exit kinetics. (D) Fixing and immunostaining for Nanog protein, to quantify the delay in transition associated with knockdown of the Tcf7l1 transcription factor by siRNA.

Different downstream tests can be performed to determine the kinetics of transition and different time points can be chosen (for examples, see Betschinger et al., 2013; Kalkan et al., 2017; Mulas et al., 2017). Assays include: (1) colony-formation assay (see ‘Colony-formation assay’ section, Fig. 5A), a key functional assay for exit of the ES cell state, which occurs asynchronously across the population, used to indicate what proportion of the population still remains responsive to naïve ES cell conditions; (2) flow cytometry using reporter cell lines (e.g. Rex1::GFPd2, Nanog-GFP, etc.; Fig. 5B,C); (3) RT-qPCR to determine the expression of genes associated with the different stages of pluripotency or differentiation; (4) fixation and immunostaining for proteins associated with ES cells (e.g. Nanog, Klf4, etc.) or transition [Oct6 (Pou3f1), Otx2, etc.] (Fig. 5D).

It is recommended to analyse multiple time points. Typically, ∼25-36 h after withdrawal of self-renewal conditions offers a time window during which both delayed and accelerated transition can be evaluated, as ∼50% of the wild-type cell population should remain responsive to 2i/LIF and positive for Rex1GFPd2. The exact timing for each experiment will depend on the starting culture condition (e.g. 2i versus 2i/LIF), the batch of N2B27, cell plating density, etc.

### Neural differentiation

#### Aim

Monolayer neural differentiation is a simple and well-characterised system (Ying et al., 2003b) and, under the right conditions, an efficient one. Therefore, it can be used to determine competence for differentiation and to examine gene expression dynamics.

#### Materials

Accutase
∼10 µg/ml laminin in PBS
Wash medium
2i (±LIF)
N2B27 (see Table S1)
Haemocytometer
Tissue culture treated plates
Falcon tubes
Centrifuge
Humidified incubator at 7% CO_2_ and 37°C

#### Standard protocol

1. Coat plates with laminin (∼10 µg/ml in PBS) overnight (ideally) or for a minimum 2 h at 37°C.
2. Aspirate laminin (do not wash) and add appropriate volume of N2B27. Return to the incubator to pre-equilibrate. Note: pre-equilibration is not necessary but it helps survival.
3. Split cells, re-suspend in N2B27 and count them.
4. Plate directly in N2B27 onto laminin-coated plate at a density of 1.0×10^4^ cells/cm^2^ or 1.2×10^4^ cells/cm^2^ for flow-sorted cells. Optional: adding 1 µg/ml laminin directly to N2B27, before plating cells in laminin-coated plates, can help with adhesion.
5. Change media to fresh N2B27 on day 2 and every day thereafter.

#### Alternative protocol for poorly adherent cells

1. Coat plates with laminin (∼10 µg/ml in PBS) overnight at 37°C.
2. Aspirate laminin (do not wash) and add appropriate volume of 2i (+LIF). Return to the incubator to pre-equilibrate. Note: pre-equilibration is not necessary but it helps survival.
3. Split cells as late as possible in the day and plate in 2i or 2i/LIF onto laminin-coated plate at a density of 1.0×10^4^ cells/cm^2^ or 1.2×10^4^ cells/cm^2^ for sorted cells. Optional: adding 1 µg/ml laminin directly into N2B27 can help with adhesion.
4. As early as possible on the next day, gently wash cells with PBS before changing the media to N2B27.
5. Change media to fresh N2B27 on day 2 and every day thereafter.

#### Notes

**Fig. 6.**
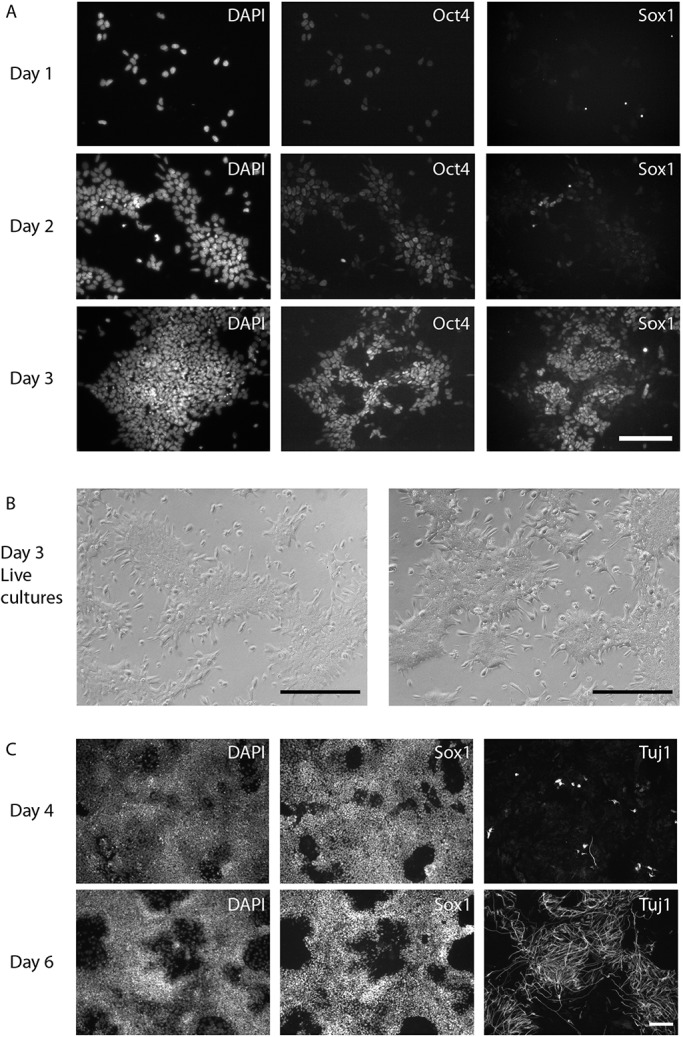
**Representative images of cells at different stages of neural differentiation.** (A) Immunostaining for Sox1 and Oct4 on day 1-3 of neural differentiation. (B) Phase contrast images showing representative morphology of early day 3 neural differentiation for two different cell lines (live cultures). Little cell death should occur during the first 1-3 days of differentiation. Cell death might become apparent once cultures become confluent. (C) Immunostaining for Sox1 and the post-mitotic marker Tuj1 on days 4 and 6 of differentiation. Scale bars: 0.5 mm.

Little or no death should be observed until cells become confluent (Fig. 6B). Significant death at day 3 is a sign of poor-quality media (see ‘Batch testing N2B27’ section) or incorrect plating density. Certain lines may require further optimisation of plating density to minimise cell death. ES cells derived from certain strains might show more cell death compared with 129 or mixed strains. When troubleshooting differentiation, we recommend plating cells at three different cell densities (e.g. 0.75×10^4^ cells/cm^2^, 1.0×10^4^ cells/cm^2^ and 1.5×10^4^ cells/cm^2^), monitoring cell death at day 3-4 and quantifying Sox1-positive cells at day 4-5. Efficient conditions typically yield ∼90% Sox1-positive cultures on day 5 with little non-neural differentiation (Fig. 6C).

The efficiency and timing of differentiation can be assessed by using the Sox1GFP reporter cell line (Ying et al., 2003b) or by measuring the expression of different neural markers such as Sox1 protein by immunofluorescence over time (Fig. 6A,C). When starting from 2i (no LIF) cells, the following changes in transcription factor expression can be anticipated: day 1, predominantly Oct4 (Pou5f1) positive, Sox2 positive, Sox1 negative; day 3, predominantly Sox2 positive (cells should be either Oct4 or Sox1 positive, as their expression is mutually exclusive), 30-50% Sox1GFP-positive cells; day 4-5, mostly Sox1-positive cells and 80-90% Sox1GFP-positive cells. Tuj1 (Tubb3)-positive cells should appear on day ∼6. Differentiation of cells cultured in 2i+LIF will be delayed by ∼1 day.

Low cell density is essential for efficient differentiation (Fig. 6A). If starting from 2i+LIF or if cells have to be pre-plated before initiating differentiation, there will be more proliferation before exiting pluripotency. Cell density can be lowered to 0.8×10^4^ cells/cm^2^.

The quality of N2B27 should be monitored. Batches should be specifically tested for neural differentiation. Certain mouse strains benefit from high insulin N2B27 (see Table S1).

Failure to change media regularly, or plating cell density higher than 1.2×10^4^ cells/cm^2^, can result in mixed differentiation (most obviously indicated by cells undergoing spontaneous contractions) and in persistence of undifferentiated ES cells.

### CRISPR/Cas9 mutagenesis

#### Aim

CRISPR/Cas9 (Cong et al., 2013; Jinek et al., 2012) allows for fast and efficient targeted mutagenesis of ES cells. As per routine ES cell culture, it is important to avoid overgrowing cells as they might develop phenotypes unrelated to the genetic perturbation. It is advantageous to use clones that go through the targeting process but that have not been edited as control cell lines. Depending on the plasmid used, transfected cells can be selected either using drug selection or by sorting for the expression of GFP.

#### Materials

Accutase
0.1-0.2% gelatine
Wash medium
FuGene
gRNA vectors
FCS (optional)
N2B27, 3 µM CHIR99021, LIF (hereafter referred to as CH/LIF; alternatively use 2i/LIF; see Table S1 for media formulation and suggested suppliers)
Penicillin and Streptomycin (PenStrep)
Puromycin (depending on the strategy)
Haemocytometer
Tissue culture treated 3.5 cm diameter plates or 12-well plates and 96-well plates for single clone picking
Falcon tubes
Centrifuge
Humidified incubator at 7% CO_2_ and 37°C
Cell sorter (depending on the strategy)

#### Transfection protocol

1. Pre-warm CL, wash medium and Accutase at 37°C. Allow FuGene to equilibrate at RT.
2. Coat 3.5 cm diameter, low-edge dish or wells of a 12-well plate with 0.1-0.2% gelatine and place in incubator for a minimum of 15 min.
3. Prepare transfection mixes as follows. Mix A: 250 ng gRNA 1, 250 ng gRNA 2, 200 μl CH/LIF (control: 200 μl CH/LIF); Mix B: 1 μl FuGene, 200 μl CH/LIF (control: 1 μl FuGene, 200 μl CH/LIF).
4. Split cells as previously indicated.
5. Just before counting cells, combine mix A and B to obtain 400 µl transfection reaction. Incubate for 5-15 min at RT (longer incubation might reduce transfection efficiency).
6. Count cells.
7. Aspirate gelatine from wells and plate 300,000 cells in 1.6 ml of CH/LIF.
8. Add 400 µl of transfection reaction to cells and mix.
9. Ensure even distribution of cells by sliding the dish across a flat surface vertically and horizontally.

#### Puromycin selection

1. The day after transfection, change media and add CH/LIF+1 µg/ml puromycin.
2. 48 h after transfection, change medium to CH/LIF+0.5 µg/ml puromycin for a further 24 h. This should kill all cells in the control plate.
3. 72 h after transfection, change medium to CH/LIF+PenStrep.
4. Day 6-8 after transfection, pick clones for expansion. The control plate should be almost completely clear. Colony number per plate ranges from 30 to 100+ depending on the gRNA.

#### GFP sorting

1. The day after transfection, change media and add fresh CH/LIF.
2. 48 h after transfection, sort single GFP-positive cells into a 96-well plate in CH/LIF+PenStrep.
3. 72 h after transfection, change medium to fresh CH/LIF+PenStrep.

#### Modifications required for generating knock-in with CRISPR/Cas9

1. Design gRNAs and clone into nickase plasmid [pSpCas9n(BB)-2A-Puro (PX462) V2.0; Addgene plasmid #62987]. The distance between the gRNA target sequences should be kept <200 bp for efficient insertion.
2. Different approaches exist for designing targeting vectors. In our hands, homology arms of 1.5 kb for the 3′ end, and 3 kb for the 5′ end work efficiently, but shorter homology arms might be used. Both gRNA target sequences should be absent in the targeting vector sequence to avoid cleavage. The amount of targeting vector should be minimised to avoid random integration.
3. A 6-well plate format should be used with a cell concentration of 1-2×10^5^ cells and a final volume of 1.6 ml before adding transfection reagent.
4. Transfection mixes should be prepared as follows. Mix A: 900 ng gRNA 1, 900 ng gRNA 2, 200 ng targeting vector, 400 μg CH/LIF; Mix B: 6 μl FuGene, 400 μl CH/LIF.

#### Single colony picking and expansion

Typically, 12-24 clones are picked per transfection and this is sufficient to obtain at least two targeted lines.

1. Coat wells of a 96-well plate (plate 1) with 0.1-0.2% gelatine in the incubator for a minimum of 15 min.
2. Pre-warm CH/LIF+PenStrep and Accutase.
3. Add 15 µl of PBS to the bottom of each well of a new 96-well plate (plate 2).
4. Under an inverted or dissection microscope in a flow cabinet, pick individual colonies and deposit in a well of plate 2 (containing PBS). If picking 36+ colonies, it is recommended to pick them in smaller batches. Do not leave colonies in PBS for more than 15-20 min. Pick colonies of different sizes. Change pipette tips between picks.
5. Add 30 µl of Accutase to each well of plate 2 and incubate for ∼5-10 min shaking occasionally.
6. Add 150 µl of CH/LIF+PenStrep to each well of plate 2 (containing the picked clones) and pipette up and down to separate colonies into single cells.
7. Aspirate gelatine from plate 1 (previously prepared).
8. Transfer all the cell suspension from plate 2 onto plate 1. Evenly disperse cells by sliding plate vertically and horizontally across a flat surface.
9. Next day, gently change media to fresh CH/LIF+PenStrep by aspirating most of the media, leaving just enough to cover cells. Take care not to detach cells.

#### Expansion of primary clones

1. Coat a sufficient number of wells in a 48-well plate with 0.1-0.2% gelatine solution for at least 15 min at 37°C.
2. Warm Accutase and CL+PenStrep at 37°C.
3. Aspirate gelatine from wells of the 48-well plate and add 200 µl of CH/LIF+PenStrep. Return plate to the incubator to pre-equilibrate.
4. Aspirate media from 96-well plates (plate 1, containing primary clones) and add 50 µl of Accutase. Incubate for ∼6 min until all the colonies have detached from the plate.
5. Add 100 µl of CH/LIF+PenStrep and pipette up and down to obtain single cell suspension.
6. Transfer 100 µl of cell suspension to the pre-equilibrated 48-well plate. Ensure uniform distribution of cells before placing in the incubator.
7. Optional: The remaining cell suspension in the 96-well plate can be used for genotyping. Add 150 µl of CH/LIF+PenStrep+1% FCS to each well of the original 96-well plate. Cells can be harvested on day 2 for genomic DNA or mRNA purification and genotyping.

#### Notes

This protocol employs SpCas9(BB)-2A-Puro (PX459) (Addgene plasmid #62988) or pSpCas9(BB)-2A-GFP (PX458) (Addgene plasmid #48138).

Set up transfections late in the day in order to be able to change media within ∼16 h post-transfection.

Each transfection will require 300,000 cells per well (round dish with 3.5 cm diameter and a low edge to facilitate colony picking). Include one extra well as a control.

Transfections in N2B27/CHIR99021/LIF (CH/LIF) result in better attachment and improved survival during selection when targeting some genes but in general 2i/LIF is effective. The addition of 1% FCS can help cell adhesion, especially after picking clones, but it is not necessary for most lines.

For efficient knockout, we routinely use two gRNA constructs per gene and FuGene HD transfection reagent (Promega, E2311). Two genes can be targeted at the same time by transfecting four gRNA constructs. In such cases, the amount of FuGene HD can be doubled.

Include a well containing only FuGene and no gRNA as a control for drug selection or cell sorting.

Do not let primary colonies overgrow as this will increase the chances of cell lines becoming compromised.

### siRNA knockdown in ES cells

#### Aim

Efficient transfection of siRNA for knockdown of single or multiple genes in cells grown in serum-free conditions.

#### Materials

Accutase
Wash medium
2i (±LIF) (see Table S1 for media formulation and suggested suppliers)
0.1-0.2% gelatine in PBS or ∼10 µg/ml laminin in PBS
Haemocytometer
Tissue culture treated 24-well plates
Centrifuge
Humidified incubator at 7% CO_2_ and 37°C

#### siRNA preparation

1. Dissolve Qiagen FlexiTube siRNAs in 50 μl RNAase-free water to obtain a 20 μM stock solution.
2. Mix equal volumes of each siRNA to obtain a 20 μM pool siRNA solution (each siRNA at 5 μM).
3. Keep on ice throughout.

#### Protocol

Set up late afternoon.

1. Coat a sufficient number of wells in 24-well plates with gelatine solution for at least 15 min at 37°C. If carrying out neural differentiation after siRNA transfection, laminin coating is recommended instead.
2. Incubate 0.5 μl pooled siRNAs in 50 µl of 2i and 0.5 μl Lipofectamine RNAiMAX (Life Technologies, 13778075) in 50 μl of 2i medium. After 2-3 min, mix and incubate for 20 min at RT (while splitting the cells).
3. Split cells and dilute to give 3.0×10^4^ cells in 400 µl of 2i for each well.
4. Aspirate gelatine from 24-well plate and combine 100 µl of siRNA solution with 400 µl of cell suspension per well.
5. Incubate overnight.
6. Next morning, wash gently with PBS and change medium and/or collect cells for RT-qPCR to determine knockdown efficiency. Note: differentiation can be initiated at this point by changing medium to N2B27.

#### Notes

**Fig. 7.**
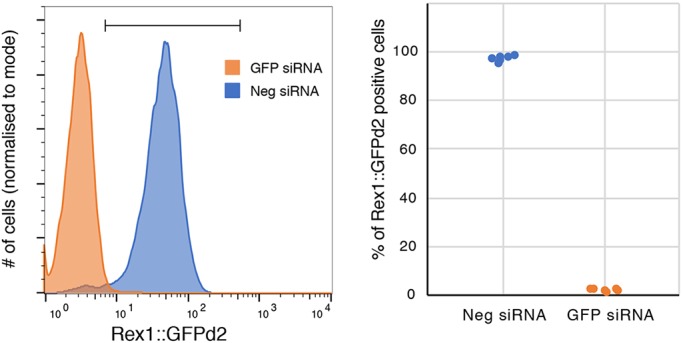
**Transfection efficiency as assessed by knockdown of GFP in Rex1::GFPd2 cells.** Left: representative flow cytometry profile. Right: quantification of the percentage of Rex1::GFPd2 positive cells over four independent experiments.

Transfect Rex1::GFPd2 cells with control or *GFP* siRNA and analyse by flow cytometry after overnight incubation. An acceptable transfection efficiency is >90% (Fig. 7). Note that this control is sensitive because GFPd2 protein is destabilised with a half-life of ∼2 h.

PenStrep- or serum-containing media cannot be used as this inhibits transfection.

Use the same media to prepare the transfection mix and culture cells (best results in 2i, without LIF).

Do not leave the cells in the transfection media for more than ∼16 h or they will die.

Cells must be actively replicating; using confluent cells (large colonies) will decrease the transfection efficiency.

Control siRNAs are as follows: GFP (custom-made), GCAAGCUGACCUGAAGUUCA; control (AllStars negative control siRNA, Qiagen, SI03650318).

### ES cell derivation

#### Aim

Derivation of ES cell lines from individual embryos, in serum-free conditions (Batlle-Morera et al., 2008; Nichols and Jones, 2017; Nichols et al., 2009b).

#### Materials

KSOM medium (Millipore, MR-106-D) or Blast (Origio, 83060010)
M2 medium (Millipore MR-015-D)
2i/LIF medium in N2B27 (see Table S1)
2i/LIF in M2
Tyrode's solution, acidic (Sigma-Aldrich T1788 or Millipore MR-004-D)
Rabbit anti-mouse antiserum (Sigma-Aldrich, M5774)
Complement sera from guinea pig, lyophilised [Calbiochem (Merck) 234395] or rat serum (as a source of complement, made in-house, not heat inactivated, kept at −80°C)
∼10 µg/ml laminin in PBS
Accutase
Dissecting microscope
Laminar flow hood
Humidified incubator at 7% CO_2_ and 37°C
Mouth pipette
Pasteur pipettes
Organ culture dishes
Tissue culture plastics

#### Protocol

1. Pre-equilibrate organ culture dishes containing KSOM (or Blast) +1 µM PDO325901 and 3 µM CHIR99021 (2i/KSOM) in the incubator for at least 15 min. Put PBS in the outer well to prevent evaporation. Flush embryos from oviducts using M2 medium at the 8-cell stage (Fig. 8A) and place into pre-equilibrated 2i/KSOM for 1-2 days until embryos reach the blastocyst stage (Fig. 8B). Note: Blast media (Origio) can be used as an alternative, but N2B27 is not suitable until after blastocyst cavitation.

**Fig. 8.**
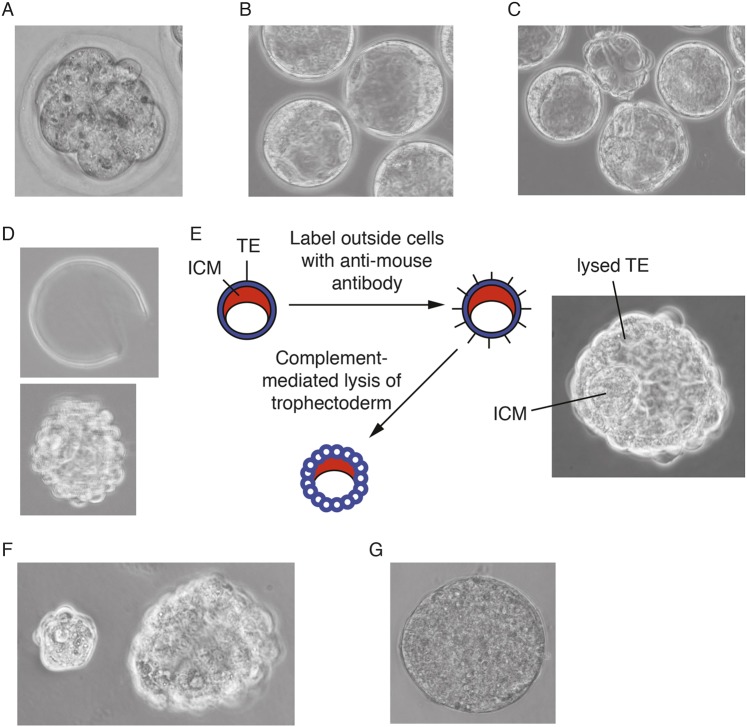
**ES cell derivation process in 2i/LIF.** (A) Flushed 8-cell embryo. (B) Embryos after 1-2 days in culture. (C) After 3 days in culture, embryos start to hatch from the zona pellucida. (D) Hatched blastocyst (bottom) and discarded zona (top). (E) Summary of immunosurgery protocol and image of ideal end-point after complement treatment. (F) Separation of ICM and trophectoderm after immunosurgery. (G) Primary outgrowth cultured for 4 days in 2i/LIF on gelatinised plates (not to scale with embryos). Images reproduced with permission from Cold Spring Harbor Laboratory Press. These images are not published under the terms of the CC-BY licence of this article. For permission to reuse, please see Nichols and Jones, 2017. TE, trophectoderm.
2. Prepare and pre-equilibrate a fresh organ culture dish containing 2i/LIF in N2B27 (LIF improves success rate, but is not essential) in the central well and PBS in the outer well.
3. Transfer embryos (now at blastocyst stage) to the new dish.
4. Incubate for 1 or 2 more days, depending on when the embryos were transferred to N2B27. A total of 3 days in culture is optimal (Fig. 8C).
5. On day 3 of culture, pre-equilibrate an organ culture dish containing N2B27+20% anti-mouse serum and three dishes containing 400 µl N2B27 in the incubator. Also, gelatinise a 96-well plate. After 20 min, aspirate gelatine and add 200 µl 2i/LIF in N2B27. Leave in the incubator to pre-equilibrate.
6. If the embryos have not hatched, remove the zona pellucida by use of acid Tyrode's solution (Fig. 8D).
7. Place a drop (∼300 µl) of Tyrode's solution on a sterile flat dish.
8. Transfer embryos still contained within the zona pellucida to Tyrode's solution with minimal carry over and monitor under a dissection microscope until the zona has been dissolved.
9. Once the zona is dissolved, wash embryos in one of the dishes of N2B27.
10. Place embryos in a pre-equilibrated dish containing N2B27+20% anti-mouse serum.
11. Incubate for 30 min to an hour or so in the incubator.
12. Rinse three times in pre-equilibrated N2B27 (or M2, if preferred) by transferring embryos through drops.
13. Add 100 µl of freshly thawed rat serum or guinea pig complement to pre-equilibrated 400 µl N2B27 to obtain a 20% solution (Fig. 8E,F). Incubate embryos in complement for ∼30 min until the trophectoderm begins to lyse (Fig. 8E). Use a Pasteur pipette of the approximate size of the inner cell mass (ICM) to remove it from the lysing trophectoderm (Fig. 8F).
14. Note: it is essential that the time between thawing and use is kept to a minimum because the complement is highly unstable.
15. Place each isolated ICM into a well of the 96-well plate with pre-equilibrated 2i/LIF in N2B27.
16. Incubate for 3-7 days, during which time each ICM will form an ES-like colony (Fig. 8G).
17. To passage primary outgrowths, gently aspirate media from wells, add 50µl Accutase per well of 96-well plate and incubate for ∼5 min until primary colonies detach. Add 100µl of 2i/LIF, pipette up and down to obtain single cell suspension, and transfer all content to a new laminin-coated well of a 96-well plate. Next day, change medium to fresh 2i/LIF. Repeat passaging procedure, progressively expanding the line to 48-well, 24-well, etc. At the 24-well stage, passage lines as indicated in ‘Propagation of ES cells without serum factors or feeders’ section.

#### Batch testing N2B27

Three main tests are carried out on batches of N2B27 to determine suitability for cell culture:

##### Colony-formation assay

See ‘Colony-formation assay’ section for a detailed protocol.

1. Culture cells in batches of 2i/LIF in N2B27 side-by-side for two or three passages.
2. For testing, coat a 12-well plate with laminin (∼10 µg/ml in PBS), three wells per batch.
3. Plate 400 cells/well in 2i/LIF+batch N2B27.
4. After 5 days, perform alkaline phosphatase staining according to manufacturer's instructions and count the number of colonies.
5. Good batches result in >80% clonogenicity.

##### Reporter assays

1. Culture Rex1::GFPd2 cells for two or three passages in 2i batch-N2B27.
2. Analyse by flow cytometry.
3. Good batches result in log-normal distributions.
4. Note: Other cell lines can be used but if the reporter protein is not destabilised (i.e. has a long half-life) cells have to be cultured for longer periods. If reporter cells are not available, immunostaining for Nanog can also be performed. Good batches result in relatively uniform Nanog expression across all cells.

##### Cell survival during differentiation

Although most batches will enable self-renewal of ES cells, differentiation requires specific attention.

1. Follow neural differentiation protocol above and examine cells on day 3-4.
2. Good batches of N2B27 show little cell death until cells reach confluence. Death on day 2-3 is evident in poor batches.

### Troubleshooting tips

Some useful troubleshooting tips are presented in Table 2.

**Table 2.**
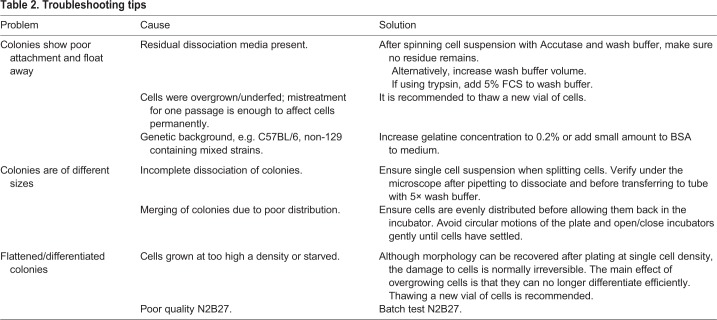
**Troubleshooting tips**

## Conclusions

In this paper, we have aimed to provide an accessible and comprehensive set of step-by-step protocols, including all media formulations, to facilitate robust and standardised manipulation of mouse ES cells in defined conditions. We provide guidelines for quality control and troubleshooting tips. The indicated reporter cell lines for calibration are available from the authors. We highlight key parameters that may perturb ES cell behaviour and genetic integrity. In particular, we emphasise the requirement for timely passaging to avoid overgrowth of colonies. Feedback on these protocols and recommendations for further improvement are welcome.

## Supplementary Material

Supplementary information
