# Correction: Defined conditions for propagation and manipulation of mouse embryonic stem cells (doi:10.1242/dev.173146)

**DOI:** 10.1242/dev.178970

**Published:** 2019-04-11

**Authors:** Carla Mulas, Tüzer Kalkan, Ferdinand von Meyenn, Harry G. Leitch, Jennifer Nichols, Austin Smith

There was an error in the supplementary data of Development (2019) 146, dev173146 (doi:10.1242/dev.173146).

Part of Table S1 was omitted from the supplementary data. Table S1 has now been updated.

The updated supplementary data are now available online at http://dev.biologists.org/content/146/6/dev173146.supplemental.

The authors apologise to readers for this mistake.

