# Supplementary material for: Defined conditions for propagation and manipulation of mouse embryonic stem cells
Source: Development. 2019 Mar 26;146(6):dev173146. doi: 10.1242/dev.173146 (PMC6451320; doi:10.1242/dev.173146)
Supplement: Supplementary information [file develop-146-173146-s1.pdf]

Table S1 - Media recipes

Three versions of N2B27 are provided. The first recipe, uses in-house N2 with added insulin (as in original N2 recipe – Ying et al. 2013) to give a final concentration of 12.5µg/ml. In the alternative versions insulin is added separately, either at 20µg/ml (which might aid neural differentiation of ES cells from certain strains, e.g. pure BL6) or at 12.5µg/ml (routine ES cell culture and differentiation). In-house preparation of N2 is recommended as commercial products vary.

Media	*Shelf-life and storage*	*Reagent*	*Supplier*	*Volume*	*Final concentration*
N2B27 – recipe for 1000ml. Can be prepared, aliquoted and stored at -80℃	<3 weeks at 4°C, can be made up to 1L, aliquoted and stored at -80°C. Avoid re-freezing.<br><br>L-Glutamine will degrade easily, avoid leaving at 37°C for long time.<br><br>Open bottles of Neurobasal should be used within a month	DMEM/F12	Sigma Aldrich - D6421	487ml	
		Neurobasal	Thermo Fisher - 21103049	487ml	
		B27 (50X)	Invitrogen, cat. no 17504044	10ml	0.5X
		N2 (with insulin* – 200X)	Made in house – aliquots at -80°C	5ml	1X
		β-mercaptoethanol (50mM stock)	Thermo Fisher - 31350-010	1ml	50µM
		L-glutamine (200mM stock)	Thermo Fisher - 25030081	10ml	2mM
Neural specific N2B27 – high insulin. Recipe for 100ml	<3 weeks at 4°C, can be made up to 1L, aliquoted and stored at -80°C. Avoid re-freezing.<br><br>L-Glutamine will degrade easily, avoid leaving at 37°C for long time.<br><br>Open bottles of Neurobasal should be used within a month	DMEM/F12	Sigma Aldrich - D6421	47.5ml	
		Neurobasal	Thermo Fisher - 21103049	47.5ml	
		B27 (50X)	Invitrogen, cat. no 17504044	1ml	0.5X
		N2.BV (200X)	Made in house – aliquots at -80°C	0.5ml	1X
		β-mercaptoethanol (50mM stock)	Thermo Fisher - 31350-010	0.1ml	50µM
		L-glutamine (200mM stock)	Thermo Fisher - 25030081	1ml	2mM
		Sodium bicarbonate (7.5% stock)	Thermo Fisher - 25080094	1.33ml	0.1%
		Optional: bovine serum albumin fraction V. (7.5% stock).	Thermo Fisher - 15260037	1.5ml	0.11%
		Insulin, human recombinant, zinc solution (4mg/ml stock)	Thermo Fisher- 12585014	0.5ml	**20µg/ml***

Development • Supplementary information

Alternative N2B27 – Recipe for 100ml. Allows using a single version of N2 (N2.BV), and adjusting the insulin concentration separately.	<3 weeks at 4°C, can be made up to 1L, aliquoted and stored at -80°C. Avoid re-freezing.<br><br>L-Glutamine will degrade easily, avoid leaving at 37°C for long time.<br><br>Open bottles of Neurobasal should be used within a month	DMEM/F12	Sigma Aldrich - D6421	47.5ml	
		Neurobasal	Thermo Fisher - 21103049	47.5ml	
		B27 (50X)	Invitrogen, cat. no 17504044	1ml	0.5X
		N2.BV (200X)	Made in house – aliquots at -80°C	0.5ml	1X
		β-mercaptoethanol (50mM stock)	Thermo Fisher - 31350-010	0.1ml	50µM
		L-glutamine (200mM stock)	Thermo Fisher - 25030081	1ml	2mM
		Sodium bicarbonate (7.5% stock)	Thermo Fisher - 25080094	1.33ml	0.1%
		Optional: bovine serum albumin fraction V. (7.5% stock).	Thermo Fisher - 15260037	1.5ml	0.11%
		Insulin, human recombinant, zinc solution (4mg/ml stock)	Thermo Fisher- 12585014	0.3125 ml	**12.5µg/ml***
Wash medium (serum-free, for accutase cultures)	~1 month at 4°C	DMEM/F12	Sigma Aldrich - D6421	500ml	
		bovine serum albumin fraction V (7.5% stock).	Thermo Fisher - 15260037	8ml	0.11%
Wash medium for trypsin dissociated	~1 month at 4°C	DMEM/F12	Sigma Aldrich - D6421	500ml	
		FCS		25ml	4.7%
2i/LIF	1 week at 4°C max. Storage at ~6-10°C will result in deterioration	N2B27	See above		
		PD0325901 (10mM stock in DMSO)	Various		1µM
		CHIR99021 (10mM stock in DMSO)	Various		3µM
		LIF (stock concentration depends on supplier)	Sigma-Aldrich L5158 or Millipore ESG1106		100U/mL
Gelatine			Sigma-Aldrich G1890		0.1-0.2% in PBS
Accutase	~2 weeks. Accutase will get deactivated if left at 37°C for a long time (~1-2hrs)		Millipore SCR005		

* Note that the original B27 recipe contained 2µg/ml insulin. The composition of commercial B27 is not available.

Development • Supplementary information
